# Prediction of drug–disease associations based on reinforcement symmetric metric learning and graph convolution network

**DOI:** 10.3389/fphar.2024.1337764

**Published:** 2024-02-07

**Authors:** Huimin Luo, Chunli Zhu, Jianlin Wang, Ge Zhang, Junwei Luo, Chaokun Yan

**Affiliations:** ^1^ School of Computer and Information Engineering, Henan University, Kaifeng, China; ^2^ Henan Key Laboratory of Big Data Analysis and Processing, Henan University, Kaifeng, China; ^3^ College of Computer Science and Technology, Henan Polytechnic University, Jiaozuo, China; ^4^ Academy for Advanced Interdisciplinary Studies, Henan University, Zhengzhou, China

**Keywords:** drug repositioning, drug-disease association prediction, graph convolutional network, metric learning, drug discovery

## Abstract

Accurately identifying novel indications for drugs is crucial in drug research and discovery. Traditional drug discovery is costly and time-consuming. Computational drug repositioning can provide an effective strategy for discovering potential drug-disease associations. However, the known experimentally verified drug-disease associations is relatively sparse, which may affect the prediction performance of the computational drug repositioning methods. Moreover, while the existing drug-disease prediction method based on metric learning algorithm has achieved better performance, it simply learns features of drugs and diseases only from the drug-centered perspective, and cannot comprehensively model the latent features of drugs and diseases. In this study, we propose a novel drug repositioning method named RSML-GCN, which applies graph convolutional network and reinforcement symmetric metric learning to predict potential drug-disease associations. RSML-GCN first constructs a drug–disease heterogeneous network by integrating the association and feature information of drugs and diseases. Then, the graph convolutional network (GCN) is applied to complement the drug–disease association information. Finally, reinforcement symmetric metric learning with adaptive margin is designed to learn the latent vector representation of drugs and diseases. Based on the learned latent vector representation, the novel drug–disease associations can be identified by the metric function. Comprehensive experiments on benchmark datasets demonstrated the superior prediction performance of RSML-GCN for drug repositioning.

## 1 Introduction

Due to the high time cost, significant investment, and laborious of the traditional drug discovery process, it is challenging to meet the needs of people facing increasingly prevalent complex diseases such as cancer, diabetes, and cardiovascular disease (Chong and Sullivan, 2007; Tamimi and Ellis, 2009). Therefore, more accurately and effectively capturing drug-related indications in drug development is of great significance. Drug repositioning, or the new use of old drugs, is an attractive means for discovering the new therapeutic potential for existing drugs that have already been approved by the Food and Drug Administration (FDA) for the treatment of diseases (Novac, 2013), so it has the advantages of reduced drug risk, a shortened clinical evaluation cycle, cost-effectiveness, and efficiency (Pushpakom et al., 2019; Luo et al., 2020). Many computational drug repositioning methods have been proposed to identify candidate indications of drugs (Lotfi Shahreza et al., 2017). These methods can be broadly classified into three major categories: (i) machine learning-based drug repositioning methods; (ii) network-based drug repositioning methods; and (iii) recommendation system-based drug repositioning methods.

Machine learning-based methods mainly utilize support vector machine (SVM) (Napolitano et al., 2013), logistic regression (Gottlieb et al., 2011; Qabaja et al., 2014), Naïve Bayes (Yang and Agarwal, 2011), and random forest (Oh et al., 2014) for classification and prediction tasks in drug repositioning. However, these traditional methods rely significantly on input data with features that have been artificially set up well to represent drug and disease characteristics, which results in a high level of implementation complexity (Yadav and Jadhav, 2019). As an extension of machine learning, deep learning has been popularly used in drug repositioning because it possesses inestimable advantages in automatically capturing nonlinear features from raw data. Zeng et al. (2019) put forward a network-based deep learning method, deepDR, which uses a multimodal deep autoencoder to learn nonlinear features of drugs from the heterogeneous networks. Network-based methods analyze the relationship between entities via message passing in different paths constructed by multiple data on the network structure, which is interpretable. Martínez et al. (2015) designed a heterogeneous network-based prioritization method to predict new drug-related diseases. Luo et al. (2016) proposed a bi-random walk (BiRW) algorithm on the drug–disease heterogeneous network to identify potential drug–disease associations. Recently, deep learning technologies have been successfully applied to drug repositioning and drug combination prediction. For example, Dehghan et al. proposed a novel multimodal deep learning-based approach called TripletMultiDTI, which incorporated multiple sources of information and used a new architecture to predict drug–target interaction affinity labels (Dehghan et al., 2022). Rafiei et al. presented a deep learning approach called DeepTraSynergy, which is designed to predict the synergistic effects of drug combinations in cancer treatment by utilizing various data including drug–target interactions, protein-protein interactions, and cell-target interactions to predict the synergistic effects of drug combinations in cancer treatment (Rafiei et al., 2023).

Recommendation system-based methods perform well in various recommend related domains including social media, e-commerce platforms, and personalized reading (Da’u and Salim, 2020). Similar to the recommendation of preferring items to users, the problem of predicting drug–disease associations can be modeled as the problem of recommending potential drugs as potential treatment to diseases (Yang et al., 2019a; Meng et al., 2022). Recently, recommended methods based on matrix factorization and matrix completion have been applied with considerable success to drug repositioning (Yang et al., 2020). Luo et al. (2018) proposed a drug repositioning recommendation system (DRRS) that uses a fast singular value threshold (SVT) algorithm (Cai et al., 2010) to fill out the unknown entries in the drug–disease adjacency matrix. Yang et al. (2019b) used the generalized matrix factorization method (GMF) involved in the collaborative filtering process to uncover the potential therapeutic relationship between drugs and diseases. Methods based on matrix factorization or matrix completion can be applied flexibly but are inefficient for large-scale data owing to complex matrix operations. In particular, the inner product operation used in the most typical matrix factorization technology violates the triangle inequality rule, potentially leading to suboptimal performance in the recommended models (He et al., 2017). In addition, this simple linear combination overlooks the modeling of the drug–drug and disease–disease relationship in a manner, and only measures the drug–disease relationship. Hence, metric learning is proposed to offset gaps in matrix factorization to enhance the expressiveness of the model. Metric learning methods have been introduced to drug repositioning in the latest studies. For instance, Luo et al. (2021) proposed a collaborative metric learning approach (CMLDR) for drug repositioning. CMLDR projected drugs and diseases into a joint metric space and then predicted the potential drug–disease pairs from the learned vectors by metric learning. While CMLDR has achieved better prediction performance, it concentrated solely on drug-centric learning to learn representations of drugs and diseases based on drug–disease association information.

Graph convolutional network (GCN) (Kipf et al., 2017) extends the convolutional neural network to solve non-Euclidean space problems. It uses structural information on the constructed network by applying convolutional operation to learn network topology preserving node-level feature embeddings to reflect complex biological entity interactions. Recently, GCN has been applied to network analysis to efficiently extract network topology feature. For drug repositioning, GCN can be utilized to extract drug and disease features from the drug-disease heterogeneous network. Then, the extracted features can be further used to calculate drug-disease association scores.

In this study, we proposed a novel computational framework for drug repositioning based on reinforcement symmetric metric learning and GCN. First, in order to alleviate the sparsity problem of drug–disease association data, we utilized Graph Convolutional Network (GCN) on drug–disease heterogeneous network to learn the features of drugs and diseases. The drug–disease association scores can be calculated based on the learned features and are used to further complement the drug–disease association matrix, which can improve the prediction performance of the model. Then, a reinforcement symmetric metric learning method with adaptive margins is proposed, which combines with drug-centric and disease-centric learning simultaneously to learn the vector representation of drugs and diseases to predict new potential drug–disease associations. Finally, we propose to integrate reinforcement symmetric metric learning and GCN model to identify potential therapeutic indications of drugs, which can provide new insights for promoting drug repositioning.

The major contributions of this study are as follows.

• This study proposed a novel framework RSML-GCN, which integrated the symmetric metric learning algorithm and GCN model to identify potential therapeutic indications for drugs, which provides insights into promoting drug repositioning.
• To relieve the problem of the sparsity of drug–disease association data, RSML-GCN applied GCN to complement drug–disease association information.
• The symmetric metric learning algorithm incorporating drug-centric and disease-centric learning is proposed to predict novel potential drug–disease associations.

## 2 Materials and methods

**FIGURE 1:**
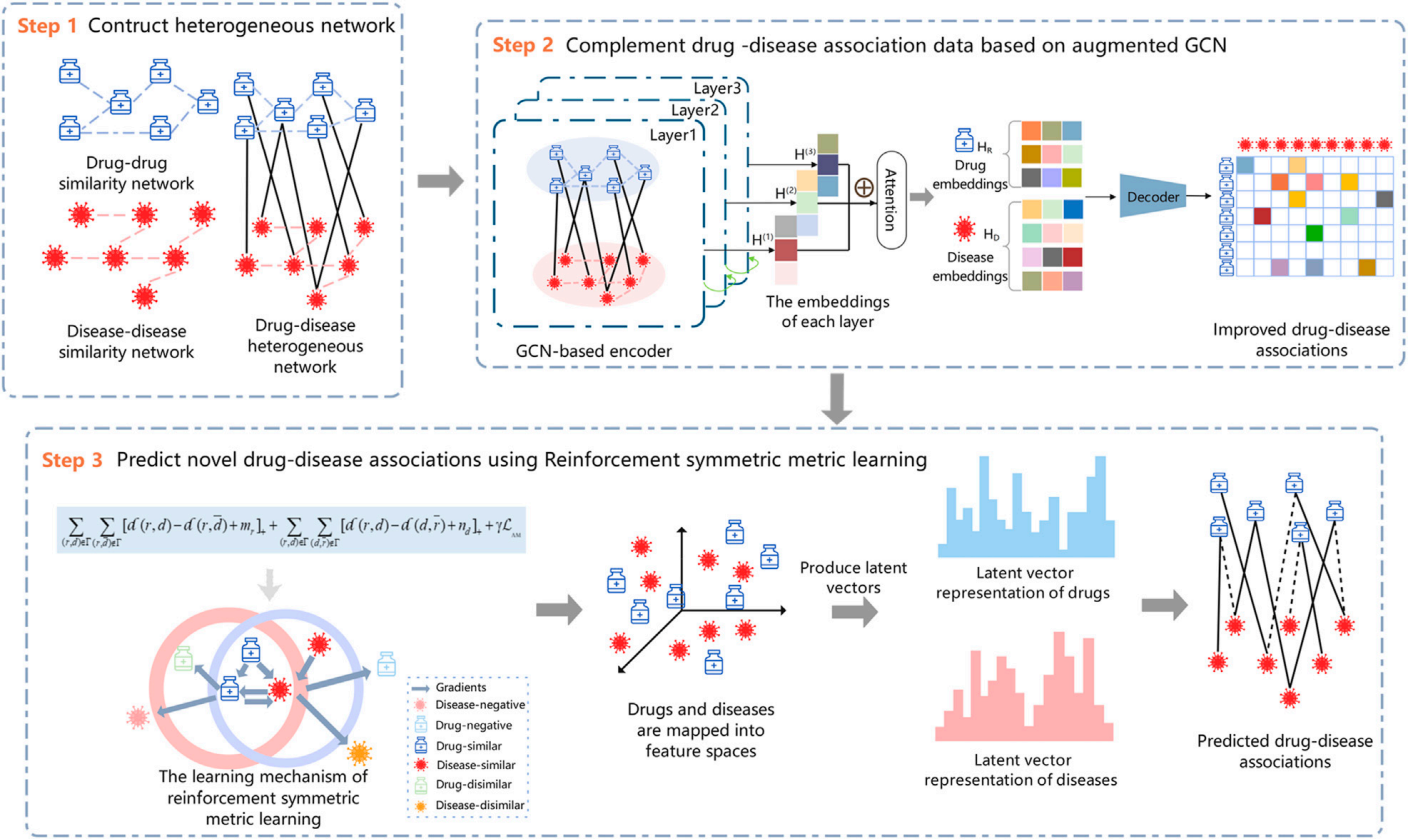
The workflow of the proposed method RSML-GCN.

In this study, we model the drug–disease association prediction as a recommendation problem and propose a new drug repositioning approach, RSML-GCN, to predict new therapies for diseases. The method combines GCN and metric learning to construct a novel framework for accurately discovering potential drug-disease associations, as shown in Figure 1. The proposed framework mainly consists of three modules including drug-disease network construction module, drug-disease complementation module and reinforcement symmetric metric learning-based prediction module. First, a drug–disease heterogeneous network is constructed based on the features and association information of drugs and diseases. Then, the low-dimensional embeddings of drugs and diseases are encoded by applying GCN, and a decoder is trained to generate an completed drug-disease association matrix by predicting drug-disease association scores. Finally, the latent representations of drugs and diseases are learned based on the reinforcement symmetric metric learning to predict novel drug-disease associations.

### 2.1 Construction of the drug–disease heterogeneous network

In this work, the similarity of drug pairs is calculated based on the Jaccard similarity coefficient, and the similarity of disease pairs is obtained by calculating the semantic similarity using medical subject descriptors.The detailed calculations are provided in Supplementary Material. A drug similarity network R and disease similarity network D can be constructed based on drug similarity and disease similarity, and the edge weight is derived from the similarity value. $A_{r}=\left\{r_{1},r_{2},\ldots ,r_{M}\right\}$ denotes the set of M drugs, and $A_{d}=\left\{d_{1},d_{2},\ldots ,d_{N}\right\}$ denotes the set of N diseases. $S_{r}\in R^{M\times M}$ denotes the adjacency matrix of the drug similarity network, and $S_{d}\in R^{N\times N}$ denotes the adjacency matrix of the disease similarity network. A drug–disease association network $S_{rd}$ can be constructed based on the known association information between drugs and diseases. An edge exists between $r_{i}$ and $d_{j}$ if there is a known association between drug $r_{i}$ and disease $d_{j}$ . The binary association matrix $Y\in {\left\{0,1\right\}}^{M\times N}$ corresponds to $S_{rd}$ , the entry $y_{ij}$ of the matrix Y is 1 if there is an edge between drug $r_{i}$ and disease $d_{j}$ , otherwise $y_{ij}=0$ which does not mean that there is no association between the drug $r_{i}$ and disease $d_{j}$ , but that there may be a potential association that has not yet been identified. For each drug $r_{i}$ , this study aims to identify diseases that are potentially associated with $r_{i}$ . The drug–disease heterogeneous network is constructed by integrating three networks: drug–drug similarity network, disease–disease similarity network, and drug–disease association network.

### 2.2 Complement drug–disease associations based on GCN

To solve the problem of the sparse verified drug-disease associations in drug repositioning, we can leverage the related information of drugs and diseases to predict potential indications of drugs to complement the drug–disease association data. GCN learns the low-dimensional representations of nodes from the irregular graph structure, and each of its layers aggregates the neighboring node information of the target node and uses the output of the previous layer as the input of the next layer, which is a process of continuously recursively aggregating neighborhood features. In this work, GCN is introduced by applying the similarity and association information to predict new drug–disease associations, which can complete the drug–disease association matrix from the biological network perspective and be used as a pre-training step to predict the likelihood of drug–disease associations.

First, the adjacency matrix G corresponding to the drug–disease heterogeneous network is defined. $S_{r}^{\prime }=E_{r}^{-1/2}S_{r}E_{r}^{-1/2}$ and $S_{d}^{\prime }=E_{d}^{-1/2}S_{d}E_{d}^{-1/2}$ are the normalized drug similarity matrix and disease similarity matrix, respectively, where $E_{r}=diag\left(\Sigma_{j}S_{r_{ij}}\right)$ and $E_{d}=diag\left(\Sigma_{j}S_{d_{ij}}\right)$ ( $S_{r_{ij}}$ or $S_{d_{ij}}$ is ( i,j )th entry of the similarity matrix) are the degree matrices of the drug and disease similarity matrices, respectively. The introduction of an appropriate degree of similarity contribution can better learn the embedding representation of drugs and diseases. Thus, a similarity penalty factor μ is introduced to control the contribution of similarity information, which can be expressed as ${\hat{S}}_{r}^{\prime }=\mu *S_{r}^{\prime }$ , ${\hat{S}}_{d}^{\prime }=\mu *S_{d}^{\prime }$ . Then, the adjacency matrix of the drug-disease heterogeneous network is represented by

$$G=\left[\begin{array}{cc} {\hat{S}}_{r}^{\prime } & Y\\ Y^{T} & {\hat{S}}_{d}^{\prime } \end{array}\right]$$ (1)

Given the matrix G , the general process of the convolution operation based on the GCN encoder according to the study of Yu et al. (2020a) can be described as

$$H^{l+1}=f\left(G,H,W\right)=\sigma \left(E^{-\frac{1}{2}}GE^{-\frac{1}{2}}H^{l}W^{l}\right)$$ (2)

Here, $H^{l+1}$ is represented as the embeddings of nodes encoded at layer l+1 , $E\left(E=diag\left(\Sigma_{j}G_{ij}\right)\right)$ is the degree matrix of the adjacency matrix G , and $H^{l}$ represents the embeddings encoded at layer l , which is used as the input at layer l+1 . W is a learnable weight matrix, and σ is a nonlinear activation function (e.g., RELU activation function).

Following the rule of Eq. 2, the GCN recursively learns node features. After l layers of iterations $\left(l=1,2,\ldots ,L\right)$ , the GCN captures information about different structures of the heterogeneous network at different layers. To enable the GCN to fully learn the features of the nodes, we use the attention mechanism to connect the embeddings of different layers of GCN learning. Different attention weights are set at different layers. The final embeddings of the obtained drugs and diseases are denoted as ${\left[\begin{array}{cc} H^{R} & H^{D} \end{array}\right]}^{T}=\sum \beta_{l}H^{l}$ . Here, $\beta_{l}$ is initialized to $1/\left(l+1\right)$ , $H^{R}\in R^{M\times k}$ and $H^{D}\in R^{N\times k}$ represent the final embeddings of the drugs and diseases, respectively.

To complement the drug–disease association matrix, we feed the final drugs and diseases embeddings into a bilinear decoder (Li et al., 2020b) for link prediction between drugs and diseases. Thus, the reconstruction of the drug–disease association matrix can be represented by $\tilde{Y}=\rho \left(H^{R}W^{\prime }H^{D^{T}}\right)$ , where ρ is the sigmoid activation function, and $W^{\prime }$ is the trainable weight matrix. Entry $y_{ij}^{\prime }$ in the matrix $\tilde{Y}$ represents the predicted score between the drug $r_{i}$ and the disease $d_{j}$ .

Ultimately, we use a binary cross-entropy loss function as the objective function to optimize the drug–disease association continuously.

$$\text{Loss}=-\frac{1}{N\times M}\left(\eta \times \sum_{\left(i,j\right)\in Y^{+}}\log y_{ij}^{\prime }+\sum_{\left(i,j\right)\in Y^{-}}\log\left(1-y_{ij}^{\prime }\right)\right)$$ (3)

where $\left(i,j\right)$ indicates the drug–disease pair, and $\eta =\left|Y^{-}\right|/\left|Y^{+}\right|$ indicates the ratio of the number of positive drug–disease pairs to the number of negative drug–disease pairs to balance positive and negative sample data.

We complement the drug–disease association information to alleviate the data sparsity problem by adopting GCN to implement pre-training on the drug–disease heterogeneous network. An entry of 1 in the drug-disease association matrix indicates that the disease is an indication for the drug and is a known association confirmed in clinical trials. In contrast, an entry of 0 means that there may be a potential association that has not yet been identified. GCN is utilized to preprocess unknown drug–disease associations to obtain more promising association information for subsequent prediction tasks. A threshold θ is set to screen highly confident drug indications. Specifically, we retain the original value if the drug–disease prediction score is greater than or equal to θ . Otherwise, we set it to 0 because a more considerable value suggests a stronger association between the drug and disease. Then, a preprocessed complemented drug–disease association matrix is obtained.

### 2.3 Reinforcement symmetric metric learning

Previous studies based on metric learning have considered drug-centric metrics (Hsieh et al., 2017; Park et al., 2018), neglecting to model drug–disease relationships from the disease perspective, which may lead to biased learning of latent vector representation of drugs and diseases, and limit the predictive performance of the model. Therefore, we take the drug- and disease-centric metrics into account for our reinforcement symmetric metric learning algorithm, which not only considers the relationships between drugs and diseases, but also implicitly establishes drug–drug and disease–disease relationships, thus enhancing the representation learning of drugs and diseases.

The goal of metric learning is to learn a metric function that pulls similar entities closer together and pushes dissimilar ones farther apart (Park et al., 2018; Wu et al., 2020). For example, when identifying possible favorite items for users in the recommendation system, metric learning assigns smaller distances to users and items with existing interactions and larger distances to users and items with unknown interactions. Similarly, it can be applied to the issue of predicting potential possible indications for drugs. The metric learning algorithms project drugs and diseases into the unified vector space and encode the latent vectors of drugs and diseases based on associations between drugs and diseases. This way, distances between drugs and diseases with known associations are closer than that between drugs and diseases without associations or with unknown associations. The likelihood of drug–disease associations is measured by the position of drugs and diseases in the unified metric vector space. Unvalidated diseases are sorted in descending order by prediction scores for a given drug, and top-k disease recommendations can be obtained.

#### 2.3.1 Problem formalization

In this work, the problem of recommending new indications for drugs is formulated as below. $A_{r}$ and $A_{d}$ denote the set of drugs and diseases, respectively, as described above. All known drug–disease associations can be designated as $\Gamma =\left\{\left(r,d\right)|r\in A_{r},d\in A_{d}\right\}$ , and $N_{i}^{+}=\left\{d_{j}|d_{j}\in \Gamma \text{ and }y_{ij}=1\right\}$ represents the set of diseases with known associations with drug $r_{i}$ . $N_{i}^{-}=\left\{d_{j}|d_{j}\notin N_{i}^{+}\text{ and }y_{ij}=0\right\}$ represents the set of diseases without known associations with drug $r_{i}$ .

Based on the completed drug–disease associations, the metric learning projects drugs and diseases into a unified n-dimensional metric vector space. In the unified metric vector space, $\alpha_{r}\in R^{n}$ is the latent vector of drug r and $\beta_{d}\in R^{n}$ is the latent vector of disease d . The association probability of drug r and disease d is measured by a simple and efficient Euclidean distance, defined as

$$d\left(r,d\right)=∥\alpha_{r}-\beta_{d}{∥}_{2}^{2},$$ (4)

where $∥*{∥}_{2}$ represents the $L_{2}$ -normalization. The calculated Euclidean distance for known drug–disease associations should be smaller than that without known associations.

#### 2.3.2 The drug-centric metric

Drug-centric metric learning is defined based on the completed drug–disease association matrix. For a given triple $\left(r,d,\bar{d}\right)$ , $\left(r,d\right)\in \Gamma$ represents a known association, which is considered a positive sample, and $\left(r,\bar{d}\right)\notin \Gamma$ represents a negative sample, which is an unknown drug–disease pair that is randomly selected. Metric learning is a similarity measure based on distance, where a closer distance means two entities are more similar. Thus, the measure of similarity can be used for the measure of correlation. Distance and correlation are two opposite concepts in drug–disease association prediction. A closer distance indicates a more possible therapeutic behavior of the drug for the disease. To ensure better learning of latent vectors of drugs and diseases, we set a margin (safe distance) m and let m>0 (Li et al., 2020a). We use the following formula to ensure that the distance between drug r and negative disease $\bar{d}$ is larger than the distance between drug r and positive disease d :

$$d\left(r,d\right)+m\leq d\left(r,\bar{d}\right)$$ (5)

**FIGURE 2:**
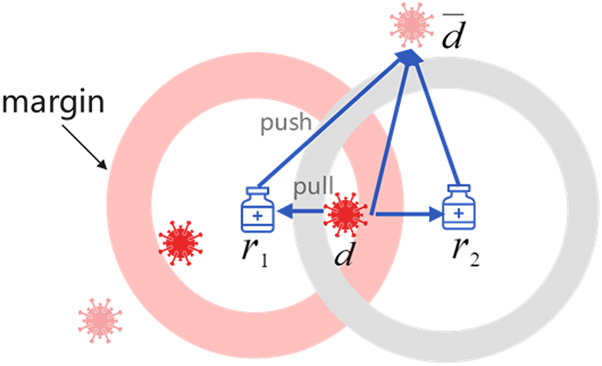
An illustration of drug-centric metric learning.

Figure 2 illustrates the drug-centric metric learning method in a two-dimensional space, where the margin is designed to separate positive and negative pairs. Specifically, drugs and diseases are represented as latent vectors in a drug–disease metric space. If the predicted drug associated with one disease, the gradient direction moves inward to limit the disease within the safe margin, otherwise, the gradient direction moves outward to keep the disease away from the drug until it exceeds the safety margin. Note that the positive disease is inside the ball centered on drug r . However, the negative disease is outside this ball centered on drug r . This guarantees that distances between drugs and positive diseases are smaller than that between drugs and negative diseases, and maximizes the correlation between drugs and associated diseases.

As a result, we adopt triple loss (Schroff et al., 2015) as the objective function for drug-centric metric learning:

$$L_{R}=\sum_{\left(r,d\right)\in \Gamma }\sum_{\left(r,\bar{d}\right)\notin \Gamma }{\left[d\left(r,d\right)-d\left(r,\bar{d}\right)+m\right]}_{+}$$ (6)

where ${\left[x\right]}_{+}=\max\left(x,0\right)$ denotes the standard hinge loss, which is a widely used loss function in the field of recommendation systems.

#### 2.3.3 The disease-centric metric

Drug-centric metric learning considers drug–disease associations from the drug perspective, thus bringing diseases associated with the targeted drug closer and having no association farther away. It is not sufficient to accurately locate the positions of drugs and diseases in the unified metric vector space to obtain their latent vectors only from the drug perspective. Moreover, drugs and diseases can be projected into the unified metric space based on the assumption that similar diseases are related to similar drugs (Xuan et al., 2019). Consequently, we introduce the disease-centric metric to explore the relationship between drugs and diseases from the disease perspective. Similarly to the drug-centric metric, for targeted disease, drugs with known associations with it are positioned close to it, or else far away. d and $\bar{r}$ are uncorrelated according to the assumption of the distance metric, so they should not be closer together and should meet $d\left(d,\bar{r}\right)>d\left(d,r\right)$ . Likewise, a margin n is set, and n>0 . The following equation is used to ensure that the distance between disease d and negative drug $\bar{r}$ is larger than the distance between disease d and positive drug r :

$$d\left(d,r\right)+n\leq d\left(d,\bar{r}\right).$$ (7)

**FIGURE 3:**
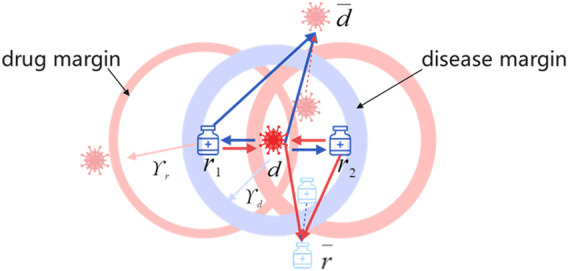
Symmetric metric learning in two-dimensional space.

Because the Euclidean distance possesses symmetry, the disease-centric learning strategy can be replaced by $d\left(r,d\right)+n\leq d\left(d,\bar{r}\right)$ . Figure 3 depicts the symmetric metric learning approach centered on drugs and diseases under the explicit treatment relationship. The disease-centric metric predicts the associated drugs from the perspective of disease and uses the safety margin for gradient learning. Obviously, the objective of symmetric metric learning is to push drugs or diseases that are not associated out of the ball and pull drugs or diseases that are associated or have potential associations into the ball. Thus, distances of known drug–disease pairs are smaller than distances between unknown pairs.

Ultimately, the objective function for the disease-centric learning is defined as below:

$$L_{D}=\sum_{\left(r,d\right)\in \Gamma }\sum_{\left(d,\bar{r}\right)\notin \Gamma }{\left[d\left(r,d\right)-d\left(d,\bar{r}\right)+n\right]}_{+}$$ (8)

In this work, we aimed to identify the relationship between drugs and diseases from the standpoint of drugs and diseases rather than directly utilizing drug-centric metric learning.

#### 2.3.4 Adaptive margin

Previous studies (Johannessen Landmark, 2008; Kingsmore et al., 2020) have found that one drug may treat multiple diseases, and that one disease may also be treated with various drugs. Considering the inconsistency of drug–disease and disease–drug association strengths, different margins are introduced for drugs and diseases. To simulate complicated drug–disease relationships better, we learn personalized margins through adaptive training. In the learning process, we set $m_{r}$ and $n_{d}$ as margins of the drug and disease, respectively. We prefer to use larger $m_{r}$ and $n_{d}$ to reduce variations. Particularly for drugs or diseases with fewer associations, more significant margins should be given to avoid overfitting, thus pushing drugs and diseases without associations farther to improve the accuracy of recommendations. Adaptive margins in the objective function can be expressed as

$$L_{AM}=-\left(\frac{1}{\left|M\right|}\sum_{r}m_{r}+\frac{1}{\left|N\right|}\sum_{d}n_{d}\right)$$ (9)

#### 2.3.5 Optimization

The number of unknown associations in the drug and disease-related data is significantly higher than the number of known associations. Therefore, we optimize the model by negative sampling. Based on known drug–disease associations, for each drug (disease), we randomly select P diseases (drugs) that are not associated with it as negative samples during the training process, and P is set as the minimum value of the number of drugs and diseases. By combining drug-centric and disease-centric metric learning losses, we obtain the final loss function for RSML-GCN as follows:

$$\begin{array}{l} L=\left(L_{R}+L_{D}\right)+\gamma L_{AM}\\ =\sum_{\left(r,d\right)\in \Gamma }\sum_{\left(r,\bar{d}\right)\notin \Gamma }{\left[d\left(r,d\right)-d\left(r,\bar{d}\right)+m_{r}\right]}_{+}+\sum_{\left(r,d\right)\in \Gamma }\sum_{\left(d,\bar{r}\right)\notin \Gamma }{\left[d\left(r,d\right)-d\left(d,\bar{r}\right)+n_{d}\right]}_{+}+\gamma L_{AM}\\ s.t.,m_{r}\in \left(0\right.,\left.l\right],n_{d}\in \left(0\right.,\left.l\right] \end{array}$$ (10)

where l is used to prevent margins from being too large to affect the performance of the prediction. Additionally, to avoid the curse of dimensionality caused by the data points spread too widely, we apply $l_{2}$ -norm clipping to the latent vectors of drugs and diseases learning, so that they are confined to the Euclidean ball with the size of l $\left({\left\|\alpha_{*}\right\|}_{2}\leq l\right.$ and $\left.{\left\|\beta_{*}\right\|}_{2}\leq l\right)$ . The objective function is then optimized by using the AdaGrad to control the learning rate to update latent vectors continuously until convergence (Duchi et al., 2011). After the training procedure is completed, Euclidean distance is used to compute the association probabilities between drugs and diseases. A complete description about the procedure of RSML-GCN is presented in Algorithm 1.

Algorithm 1 RSML-GCN Algorithm.

**Input:** The matrix of known drug–disease associations $Y\in {\left\{0,1\right\}}^{M\times N}$ ; The drug similarity matrix $S_{r}\in R^{M\times M}$ ;The disease similarity matrix $S_{d}\in R^{N\times N}$ ; Hyper parameters k , L , $lr_{1}$ , n , $lr_{2}$ and γ .
**Output:** The predicted drug-disease association matrix $\hat{Y}$ .
**1:** Normalize drug similarity matrix $S_{r}^{\prime }$ and normalized disease similarity matrix $S_{d}^{\prime }$ and initialize drug–disease heterogeneous graph G .
**2:** repeat
**3:**    **for** l=1,2,…,L **do**
**4:**       Learn node features $H^{l}$ with Eq. 2;
**5:**    **end for**
**6:**      Combine nodes embeddings $H^{l}$ with $\sum \beta_{l}H^{l}$ , obtain the final embeddings of drugs $H^{R}$ and the final embeddings of diseases $H^{D}$ ;
**7:**    Obtain the prediction matrix $\tilde{Y}$ with $\rho \left(H^{R}W^{\prime }H^{D^{T}}\right)$ ;
**8:**    Update parameters by optimizing Eq. 3;
**9:** until Eq. 3 is converged, get $\tilde{Y}$ ;
**10:** ${\tilde{Y}}^{*}$ is obtained by screen $\tilde{Y}$ using a threshold θ ;
**11: for** $\left(r,d\right)\in$ sampled drug–disease associations in ${\tilde{Y}}^{*}$ **do**
**12:**   sample a negative drug–disease $\left(r,\bar{d}\right)$ pairs to build a triplet $\left(r,d,\bar{d}\right)$ ;
**13:**   Compute $L_{R}$ with Eq. 6;
**14:**   sample a negative disease-drug $\left(d,\bar{r}\right)$ pairs to build a triplet $\left(d,r,\bar{r}\right)$ ;
**15:**   Compute $L_{D}$ with Eq. 8;
**16: End for**
**17: While** not converged **do**
**18:**   Compute gradients;
**19:**   Update $\alpha_{r}$ and $\beta_{d}$ with AdaGrad on Eq. 10;
**20:**   Compute the predict probability;
**21:** $P=∥\alpha_{r}-\beta_{d}{∥}_{2}^{2}$ ;
**22:**   Check whether the model converges on the validation set;
**23: End while**
**24:** $\hat{Y}=P$ ;
**25: Return** $\hat{Y}$ ;

## 3 Results and discussion

### 3.1 Comparison with other methods

To verify the effectiveness of our method in predicting drug–disease associations, we compared RSML-GCN with five state-of-the-art drug repositioning methods based on recommendation system and GCN including GRGMF (Zhang et al., 2020), DRWBNCF (Meng et al., 2022), LAGCN (Yu et al., 2020b), DRHGCN (Cai et al., 2021) and CMLDR (Luo et al., 2021). These methods are detailed below.

• GRGMF establishes a generalized matrix factorization model that obtains the latent representation of each node by adaptively learning the neighborhood information of each node, and it introduces external similarity information to facilitate the prediction of potential links.
• DRWBNCF is a neural collaborative filtering method that proposes a new weighted bilinear graph convolution operation to integrate the information of the known drug–disease association, drug’s and disease’s neighborhood, and neighborhood interaction into a unified representation to infer novel potential drug–disease associations.
• LAGCN is a layer attention GCN that uses GCN to learn embeddings of drugs and diseases from the drug–disease heterogeneous network. The learned embeddings are then integrated by an attention mechanism to predict new associations.
• DRHGCN uses GCN to extract inter-domain and intra-domain feature information of drugs and diseases to find new drug indications based on different network topology information of drugs and diseases in different domains.
• CMLDR is a collaborative metric learning algorithm that predicts the association probability of drugs and diseases by applying metric learning. The latent vectors of drugs and diseases are learned based on the known related information of drugs and diseases and used to identify candidate drug–disease associations.

For a fair comparison, we ran these competing methods with the optimal parameters suggested in the original papers on benchmark datasets. The complete evaluation of all methods was performed under 10-fold cross-validation. The specific experimental settings are described in Supplementary Material. Also, we conducted parameter analysis and selected the best parameters as the recommended settings for RSML-GCN in this work.

### 3.2 Parameter setting

Considering that hyperparameters could affect model performance, we further investigate the influence of hyperparameters including that used in GCN, such as the latent vector dimension n , the marginal value strengths γ , and weight variables. The specific hyperparameter settings are given in Supplementary Material. According to the previous study (Yu et al., 2020a), we set the parameters for GCN with the embedding dimension k=64 , number of layers L=3 , initial learning rate $lr_{1}=0.008$ , node discard rate β=0.6 , regularize discard rate ξ=0.4 , and penalty factor μ=6 . Moreover, we have investigated the effect of the latent vector dimension *n* by varying its value from 30 to 400, and examined the influence of the marginal value strengths γ by varying its value from 0.01 to 100. The optimal parameters were determined by the grid search method, and detailed information is provided in the Supplementary Material. Finally, the latent vector dimension of drugs and diseases in the metric space was fixed at 250, the initial learning rate $lr_{2}$ was 0.05, and the batch size was 512. In terms of variables, refer to the settings of Li et al. (2020a), all weight variables followed a uniform distribution [-0.01, 0.01] and were randomly initialized, and all latent vectors (such as $\alpha_{r}$ , $\beta_{d}$ ) that follow a normal distribution (mean: 0.1, variance: 0.03) were randomly initialized. More detailed parameter settings are described in Supplementary Figures S1–S4.

### 3.3 Performance of RSML-GCN in cross-validation

To evaluate the performance of RSML-GCN, we conducted extensive experiments on two benchmark datasets Cdataset and Fdataset in Supplementary Table S1 and compared RSML-GCN with five state-of-the-art association prediction methods. The performance evaluation results of all methods under 10 times 10-fold cross-validation were reported in Table 1. The experimental results show that RSML-GCN had good performance in relevant metrics and was superior to other methods. In terms of the primary metric, AUPR, RSML-GCN achieved the highest average value of 0.7941, which surpasses GRGMF by 33.7%, and the average AUPR values of DRWBNCF, LAGCN, DRHGCN and CMLDR were 0.4992, 0.1562, 0.5480, and 0.2607, respectively. Additionally, RSML-GCN outperformed other methods in terms of AUC, with an average AUC value of 0.9077. This was 0.20% higher than the second-best method, DRHGCN. DRWBNCF, GRGMF, LAGCN and CMLDR have AUCs of 0.8642, 0.8994, 0.7874 and 0.7999, respectively.

**TABLE 1:** Results of different methods under 10 iterations of 10-fold cross-validation.

Datasets	DRWBNCF	GRGMF	LAGCN	DRHGCN	CMLDR	RSML-GCN
AUPR						
Cdataset	0.4821	0.5611	0.1946	0.5562	0.1088	0.8580
Fdataset	0.5163	0.6269	0.1178	0.5397	0.4125	0.7302
Avg	0.4992	0.5940	0.1562	0.5480	0.2607	0.7941
AUC						
Cdataset	0.8480	0.8638	0.8358	0.8756	0.7650	0.9309
Fdataset	0.8803	0.9350	0.7389	0.9362	0.8348	0.8846
Avg	0.8642	0.8994	0.7874	0.9059	0.7999	0.9077

We have performed 10 times 10-fold cross-validation and obtained AUC and AUPR values for all methods. The paired *t*-test is applied to statistically test the significance between the proposed method and other existing methods in terms of AUPR values, which have been conducted in previous studies. The paired *t*-test results including the *p*-values are showed in Table 2. It can be observed that RSML-GCN is statistically significantly better than other methods (*p* < 0.05).

**TABLE 2:** The statistical significance of performance improvements achieved by RSML-GCN.

Paired *t*-test	Fdataset	Cdataset
RSML-GCN vs. DRWBNCF	8.44E-25	4.11E-29
RSML-GCN vs. GRGMF	3.92E-22	2.48E-32
RSML-GCN vs. LAGCN	1.81E-23	2.0E-28
RSML-GCN vs. DRHGCN	4.36E-17	4.83E-33
RSML-GCN vs. CMLDR	5.11E-29	2.48E-39

The drug–disease prediction problem was formulated as a top-k recommendation problem, where potential therapeutic diseases are recommended for a specific drug. Therefore, we used top-k prediction results as evaluation metrics, specifically precision@K (p@K) and recall@K (r@K), which are widely used in recommendation domains. The performance of different models in predicting the top-k drug–disease associations on Cdataset was reported in Supplementary Figure S5. RSML-GCN outperformed other models in terms of r@5, r@10, p@5, and p@10. Additionally, in Supplementary Figure S6, we can find that RSML-GCN also achieves excellent performance in the recall and precision values of the top-k predictions on Fdataset, which is much better than collaborative filtering-based, GCN-based, and metric learning-based methods. Notably, the performance indicators of LAGCN in these results were inferior to those of other methods, potentially due to GCN exhibiting over-smoothing issues stemming from dataset imbalances. The prediction results of the matrix factorization method GRGMF were lower than RSML-GCN, indicating that the metric learning method can effectively compensate for the shortcomings of matrix factorization. In contrast, CMLDR yielded significantly lower results than our proposed method, which suggests the usefulness of increasing the disease-centric auxiliary reuse learning for improving the drug-centric metric. The superior performance of RSML-GCN can be attributed to the following aspects. First, deep learning method is utilized to learn the potential representations of drugs and diseases and generate high confident drug–disease associations. This effectively alleviates the sparsity problem of drug–disease association data and improves the performance of subsequent task predictions. Second, we designed a reinforcement metric learning method to learn the metric between drugs and diseases from both drug and disease aspects, which can improve previous metric learning methods. Finally, by integrating the deep learning method and metric learning method, the proposed method can achieve better performance than other drug–disease prediction methods. Furthermore, we have avoided excessive integration of biological data, as improper handling of such data can introduce noise and adversely affect prediction results. These results comprehensively demonstrate the effectiveness of our proposed method in identifying drug–disease associations.

### 3.4 Ablation experiment

To evaluate the model performance of RSML-GCN, we set up a variant of RSML-GCN, named as RSML. In RSML, we used only reinforcement symmetric metric learning to predict drug–disease association scores, which removes the pre-training step of complementing the drug–disease association matrix using GCN. In order to check the contribution of the pre-training component, we compared RSML-GCN with RSML based on Cdataset.

Based on the drug–disease association matrix, the RSML projected drugs and diseases to the unified metric vector space and learned their latent vectors based on the push–pull mechanism. The Euclidean distance was adopted to obtain the potential treatment probabilities of drugs for diseases. As can be seen in Supplementary Table S2, incorporating GCN in RSML-GCN as a pre-training step to complement the drug–disease association matrix resulted in improved predictive performance. The average AUPR of RSML-GCN was 6.45% higher than that of RSML, while maintaining a comparable AUC. Additionally, significant enhancements were observed across all top-k prediction evaluation metrics, as depicted in Supplementary Figure S7. This improvement can be attributed to GCN’s ability to integrate similarity information from drug–disease associations, enabling the learning of more comprehensive representations and acquiring more confident drug–disease association information. Consequently, this approach helps address the imbalance between positive and negative samples to serve downstream tasks better and improve the predictive potential of metric learning method. The results generally indicate the reliability of RSML-GCN for predicting drug-related diseases.

### 3.5 Predicting candidates for new drugs or new diseases

To assess the ability of RSML-GCN in predicting potential indications for new drugs, we removed the associated diseases of the test drug and predicted indications for it on Cdataset. To more accurately display the top-k recommendation performance of the model, we selected drugs associated with at least 50 diseases to evaluate the performance of RSML-GCN for new drug prediction. After training, the latent vectors of drugs and diseases in the training samples were learned. For a new drug without any known association, RSML-GCN could obtain latent vectors of the drug by utilizing similarity information from its h-nearest neighbors in the training set to predict the potential drug-related diseases. In the experiment, empirically, h was set to 5 to simplify the model.

**FIGURE 4:**
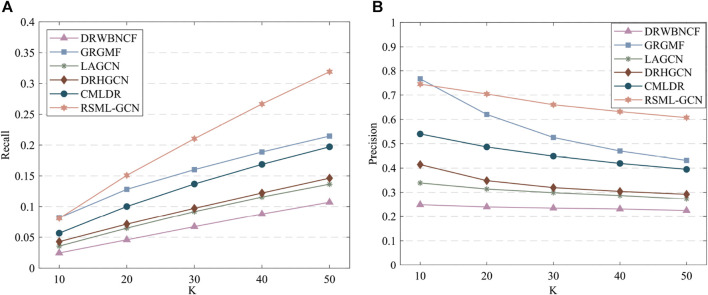
The recall values **(A)** and precision values **(B)** of various methods in predicting top-k diseases new drugs.

The results of predicting unknown diseases for new drugs are presented in Supplementary Table S3, RSML-GCN exhibited the best performance in the primary metric AUPR (average AUPR = 0.5555), which is higher than GRGMF and CMLDR based on recommendation system by 49.0% and 74.4% (AUPR value), respectively. In terms of AUC, RSML-GCN had an average AUC of 0.6985, which is higher than that of these state-of-the-art prediction methods. The recall and precision of top-k recommendations of RSML-GCN for predicting potential indications for new drugs were reported in Figure 4, which shows the performance of RSML-GCN over other methods for different values of K. For the average recall value, our RSML-GCN performed better than other methods under most K values. For example, when K = 10 and K = 50, RSML-GCN achieved the best average recall values, 0.0807 and 0.3191, respectively. In particular, when K = 10, DRWBNCF, LAGCN, DRHGCN, and CMLDR obtained recall values of 0.0245, 0.0356, 0.0428 and 0.0565, respectively, the recall values of GRGMF and RSML-GCN were almost comparable. In addition, when K = 10 and K = 50, RSML-GCN attained average precision values of 0.7451 and 0.6072, respectively, which is higher than most competitive methods. Overall, the comprehensive results demonstrate that RSML-GCN has an excellent ability to predict related diseases for new drugs.

For a new disease without any known associations, RSML-GCN can use the similarity information of diseases to predict potential candidate drugs for new diseases. We also conducted the experiments, in which all relationships for each disease were removed to predict candidate drugs for new diseases. The results compared with state-of-the-art methods were reported in Supplementary Table S4 and Supplementary Figure S8. RSML-GCN was the second-best, significantly better than DRWBNCF, LAGCN, DRHGCN, and CMLDR. The recall and precision of RSML-GCN also achieved the second-best performance. The reason is that the input of GRGMF contains both drug–drug similarity and disease–disease similarity, while the input of RSML-GCN only contains known drug–disease associations.

### 3.6 Independent test experiments

**FIGURE 5:**
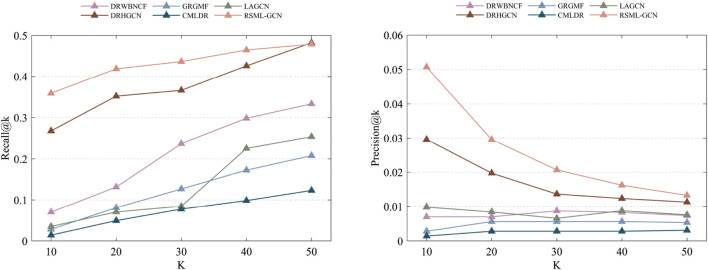
The recall and precision values of the top-k recommended drug–disease associations are achieved by different methods on the independent test set.

We also investigated the performance of these prediction methods on the independent test set, another dataset released by Luo et al. (2016) is used to assess the performance of methods. By removing the drugs not included in Fdataset, we obtained an independent test set consisting of 89 drug–disease associations involving 71 drugs and 313 diseases. This test set was used to assess the performances of all prediction methods in predicting the drug–disease associations on the Fdataset. Overall, the performance of all the methods moderately deteriorates relative to the 10-fold cross-validations. RSML-GCN remained the best method, which achieved an AUPR value of 0.3030 and an AUC value of 0.6842. DRWBNCF and LAGCN achieved AUC values of 0.6218 and 0.6215, respectively (Table 3). We also show the ability to correctly predict drug–disease associations concerning given top-k thresholds, as shown in Figure 5. Accordingly, RSML-GCN can predict drug–disease associations more accurately than all other five methods on almost every top-rank threshold.

**TABLE 3:** Results on independent test set.

Methods	DRWBNCF	GRGMF	LAGCN	DRHGCN	CMLDR	RSML-GCN
AUPR	0.0353	0.0140	0.0220	0.0520	0.0459	0.3030
AUC	0.6218	0.5313	0.6215	0.7783	0.5355	0.6842

### 3.7 Case study

In this section, we conducted a case study to further evaluate the reliable ability of RSML-GCN to predict novel drug–disease associations. For the analysis, we chose three representative drugs for the treatment of high-incidence diseases, Atorvastatin Calcium, Etoposide, and Riluzole. Atorvastatin Calcium is a commonly used lipid-lowering drug in the clinic, which is mainly used to treat mixed hyperlipidemia and hypercholesterolemia (Egom and Hafeez, 2016). These diseases have a high incidence, are difficult to diagnose and treat, and can potentially induce Cardio-cerebrovascular disease (Yao et al., 2019). Therefore, the analysis of Atorvastatin Calcium is of great significance. Etoposide is a cell cycle specific antitumor drug that is primarily effective against small cell lung cancer (Mascaux et al., 2000), acute leukemia, and malignant lymphoma. Given cancer is complicated and difficult to cure, it is valuable to analyze whether Etoposide can treat other similar diseases in drug reuse. Riluzole is a central nervous system drug that plays a pivotal role in the treatment of Alzheimer’s disease, Parkinson’s disease, and brain injury, which have a serious impact on patients. Therefore, it is necessary to analyze the new therapeutic potential for this drug to treat a variety of neurological degenerative diseases. Specifically, we applied RSML-GCN to predict candidate diseases for three drugs. For each of the three drugs, all predicted candidate disease scores were ranked by priority, and then we excluded all known drug–disease associations from the primary dataset to generate a new top-ranked list of drug–disease associations. Finally, we used highly reliable sources and clinical trials (i.e., DrugBank (DB) (Law et al., 2013), CTD (Davis et al., 2016), PubChem (Kim et al., 2015), DrugCentral (Avram et al., 2020), and ClinicalTrials) as references to examine the predicted drug–disease associations. Table 4 presents the predicted results of the top 10 candidate diseases for three drugs. The results show Atorvastatin Calcium can also be shown to treat lung disease, left ventricular dysfunction, and is also associated with kidney failure, which are supported by CTD, ClinicalTrials, and DrugCentral. The discovery of Etoposide can be verified in all clinical trials, which shows that Etoposide not only has a good therapeutic effect on a variety of tumors but also can be used to treat Exanthema and drug eruption. In addition, Riluzole was also found to be related to heart failure, drug-induced liver injury, and arrhythmia. To sum up, most of our predictions can be verified by reliable sources and clinical trials. The case study results further demonstrate the effectiveness of RSML-GCN in predicting novel drug–disease associations.

**TABLE 4:** The top-10 candidate diseases predicted by RSML-GCN for three drugs.

Drug	Rank	Disease	Evidences	Rank	Disease	Evidences
Atorvastatin Calcium	1	Liver Diseases	CTD/ClinicalTrials/DrugCentral	6	Headache	CTD
	2	Ventricular Dysfunction, Left	CTD	7	Hyperalgesia	CTD
	3	Liver Neoplasms	CTD/ClinicalTrials/DrugCentral	8	Renal Insufficiency	CTD/ClinicalTrials/DrugCentral
	4	Vomiting	CTD	9	Edema	CTD/ClinicalTrials
	5	Dizziness	NA	10	Weight Gain	CTD
Etoposide	1	Exanthema	CTD	6	Carcinoma, Squamous Cell	CTD/ClinicalTrials
	2	Drug Eruptions	CTD	7	Skin Neoplasms	CTD/ClinicalTrials
	3	Uterine Cervical Neoplasms	CTD/ClinicalTrials	8	Leukemia	CTD/ClinicalTrials/DB/PubChem/DrugCentral
	4	Carcinoma, Transitional Cell	CTD/ClinicalTrials	9	Lung Diseases, Interstitial	CTD/ClinicalTrials/DrugCentral
	5	Lymphoma, Large	CTD/ClinicalTrials/DB/PubChem/DrugCentral	10	Cerebellar Diseases	CTD/ClinicalTrials
Riluzole	1	Heart Failure	CTD	6	Drug-Related Side Effects and Adverse Reactions	CTD
	2	Chemical and Drug Induced Liver Injury	CTD/DrugCentral	7	Myocardial Infarction	CTD
	3	Acute Kidney Injury	CTD	8	Hypotension	CTD/ClinicalTrials
	4	Arrhythmias, Cardiac	CTD/ClinicalTrials	9	Rhabdomyolysis	NA
	5	Kidney Diseases	NA	10	Brady-cardia	CTD

## 4 Conclusion

In this study, we proposed a new framework for drug–disease association prediction by incorporating GCN and reinforced symmetric metric learning, named RSML-GCN. Firstly, in order to alleviate the sparsity problem of drug–disease association data, the GCN was applied to capture the structure of network topology on the heterogeneous network constructed by the biological knowledge and known association information of drugs and diseases to complement the missing drug–disease association information, which improves the prediction performance of the model. Secondly, the current metric learning algorithm only learns in a single way centered on drugs, ignoring the influence of diseases. Therefore, a reinforcement symmetric metric learning algorithm combined with drug-centric and disease-centric learning was developed to project drugs and diseases into a unified metric space, and learn their latent vector representations based on push–pull mechanisms to identify potential indications for known drugs and new drugs. Based on the assumption that similar drugs can treat similar diseases, the disease-centric metric learning mechanism was introduced symmetrically, which improved on the previous approach. Moreover, the adaptive margin strategy helped the model select the appropriate margin for different drugs and diseases. Thirdly, this study proposes a new framework integrating reinforcement symmetric metric learning algorithm and GCN model to identify potential therapeutic indications of drugs, which provides new insights for promoting drug repositioning. The results of extensive experiments demonstrated that RSML-GCN performed well and outperformed other drug–disease association prediction methods.

RSML-GCN only utilized drug–disease association data and the single feature information of the drug and the disease to predict potential associations. However, there exists various drug and disease related biological data, and the use of multiple data may help to learn potential indications for drugs. Therefore, in the future of work, more biological data including genes, targets, or miRNAs can be considered and integrated to build a more comprehensive heterogeneous network with multiple relationship types. In addition, the metric learning algorithm only uses known drug–disease association information as input. Future research should design an effective way to integrate related biological data into its learning process to predict potential drug–disease associations.

## Data Availability

The original contributions presented in the study are included in the article/Supplementary Material, further inquiries can be directed to the corresponding author.
