# Supplementary material for: Prediction of drug–disease associations based on reinforcement symmetric metric learning and graph convolution network
Source: Front Pharmacol. 2024 Feb 7;15:1337764. doi: 10.3389/fphar.2024.1337764 (PMC10879308; doi:10.3389/fphar.2024.1337764)
Supplement: Supplementary file 1 [file DataSheet1.PDF]

Supplementary Material

Prediction of drug–disease associations based on reinforcement symmetric metric learning and graph convolution network

Huimin Luo, Chunli Zhu, Jianlin Wang Ge Zhang, Junwei Luo and Chaokun Yan*

*** Correspondence:** Corresponding Author: ckyan@ henu.edu.cn

1 Supplementary Methods

1.1 Measure drug–drug similarity

According to the target feature descriptor of the drug, a drug can be encoded as a binary feature vector, where a 1 in the vector indicates that the drug is associated with the corresponding target, and a 0 indicates that the drug is not associated with the corresponding target. Based on the target features of the drug, we calculate drug–drug similarities with the help of the Jaccard similarity coefficient (Deng et al., 2020). The two binary feature vectors α_i and α_j denote the target features of drug i and drug j, respectively, whose Jaccard similarity coefficients can be determined by

$$S_{ij} = \frac{|\alpha_i \cap \alpha_j|}{|\alpha_i \cup \alpha_j|} \tag{1}$$

where $|\alpha_i \cap \alpha_j|$ denotes the number of elements that are 1 in the corresponding positions of α_i and α_j, and $|\alpha_i \cup \alpha_j|$ denotes the number of elements that are 1 in either the elements of α_i or the corresponding ones of α_j. $S_{ij} \in [0,1]$ denotes the similarity value between drug i and drug j.

1.2 Measure disease–disease similarity

We can use the MeSH information to measure the semantic similarities between two diseases. Each disease can be viewed as a hierarchical directed acyclic graph (DAG) (Guo et al., 2020), in which nodes represent disease MeSH descriptors, and edges represent the relationship between the current node and its ancestor nodes. For a disease O, its DAG consists of disease O and all its ancestor diseases, which can be represented by $DAG(O) = (N(O), E(O))$, where $N(O)$ is the set of all ancestor nodes of O (including itself), $E(O)$ is the set of edges for which O has all relationships with its ancestors. The semantic value $C_O(d)$ that a disease d in $N(O)$ contributes to disease O is defined by the following formula:

$$C_O(d) = \begin{cases} 1 & if\ \ d = O \\ max\{\lambda * C_O(d') \mid d' \in children\ of\ d\} & if\ \ d \neq O \end{cases} \tag{2}$$

where $\lambda \in [0,1]$ is a contribution factor, as in the study by Wang et al. (2010), λ is set to 0.5, and the total semantic value of disease d in $N(O)$ contributing to disease O is defined as $DV(O) = \Sigma_{d \in N(O)} C_O(d)$.

According to the hypothesis that diseases with more common ancestors in the DAG tend to have higher semantic similarity, the semantic similarity between disease O and P is defined as follows:

$$S_{O,P} = \frac{\sum_{d \in N(O) \cap N(P)} \left(C_O(d) + C_P(d) \right)}{DV(O) + DV(P)}, \tag{3}$$

where $C_P(d)$ is the semantic value of disease d related to disease P, and $DV(P)$ is the total semantic value of disease d's contribution to disease P.

1.3 Experimental settings

In this study, we conducted 10-fold cross-validation to evaluate the performance of the model. More concretely, all the known drug–disease associations that had been verified were randomly divided into 10 approximately equal subsets. Each subset was taken in turn as test data, the remaining nine subsets were used as the training data, and 10% of the training data were randomly selected as the validation data. In each fold, a prediction model was built on the training data based on the known associations, its parameters were adjusted using the validation data, and then the prediction was implemented on the test data. To ensure that the results of 10-fold cross-validation were unbiased, we carried out 10 independent 10-fold cross-validation experiments, and the average results were used to measure the model performance.

To assess the accuracy of the RSML-GCN model, we used the receiver operating characteristic curve (ROC), which was plotted from two variables, the false positive rate and the true positive rate, resulting in the area under the curve (AUC) value, which can be applied to binary classification issues and has been extensively used in prior research (Wu and Flach, 2005). Because AUC cannot fully generalize the model performance, we presented a second evaluation metric, AUPR, which leverages the precision-recall (PR) curve to accurately reflect the actual performance of the prediction model (Zhao et al., 2021). There are far more drugs and diseases with no association than those with known drug–disease associations. Here, AUPR was used as the primary metric for such class-imbalanced datasets (Flach and Kull, 2015). As the number of correctly predicted true positives reflects the ability of the model to identify positive and negative samples, especially when the number of positive samples is much smaller than the number of negative samples, Precision and Recall were also employed as indicators of model effectiveness.

2 Supplementary Tables and Figures

2.1 Supplementary Tables

Supplementary Table 1. Statistics of the dataset. (In this study, we adopted two benchmark datasets to evaluate the performance of RSML-GCN. The first one is Cdataset, which corresponds to the gold standard dataset used in the work of (Zhang et al., 2018). The verified drug–disease association data is derived from the Comparative Toxicogenomics Database (CTD) (Davis et al., 2016), which is a publicly available database containing interaction information between drugs, diseases, genes, and functional phenotypes. The Cdataset contains 269 drugs and 598 diseases, with 18 416 drug–disease pairs with proven associations. In addition, drug-target information is collected from the DrugBank

database (Law et al., 2013), which is used as the characteristics of drugs. The characteristics of diseases are defined based on medical subject headings (MeSH), a subject glossary compiled by the National Library of Medicine as biomedical indexing, which provides semantic feature descriptors for diseases (Lipscomb, 2000). Therefore, we use Cdataset to comprehensively test the performance of our method. The second dataset Fdataset (Gottlieb et al., 2011) contains 1933 known drug–disease associations between 593 drugs obtained in DrugBank database and 313 diseases extracted from OMIM database.)

Dataset	Drugs	Diseases	Number of association
Cdataset	269	598	18416
Fdataset	593	313	1933

Supplementary Table 2. Comparison of results of ablation experiments under 10 iterations of 10-fold cross-validation.

Methods	RSML	RSML-GCN
AUPR	0.8060	0.8580
AUC	0.9220	0.9308

Supplementary Table 3. AUPR and AUC performance of different methods in predicting diseases for new drugs.

Methods	DRWBNCF	GRGMF	LAGCN	DRHGCN	CMLDR	RSML-GCN
AUPR	0.2105	0.3727	0.2704	0.2599	0.3186	0.5555
AUC	0.5382	0.6649	0.5572	0.6037	0.6424	0.6985

Supplementary Table 4. AUPR and AUC performance of different methods in predicting drugs for new diseases.

Methods	DRWBNCF	GRGMF	LAGCN	DRHGCN	CMLDR	RSML-GCN
AUPR	0.3189	0.6890	0.5368	0.5373	0.6056	0.6196
AUC	0.5239	0.8114	0.7287	0.7386	0.7771	0.7950

2.2 Supplementary Figures

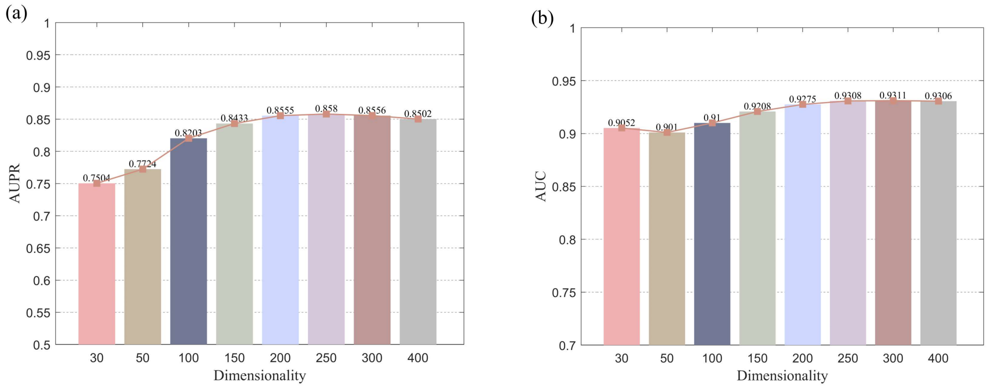

Supplementary Figure 1. The AUPR and AUC of RSML-GCN with different dimensional settings.

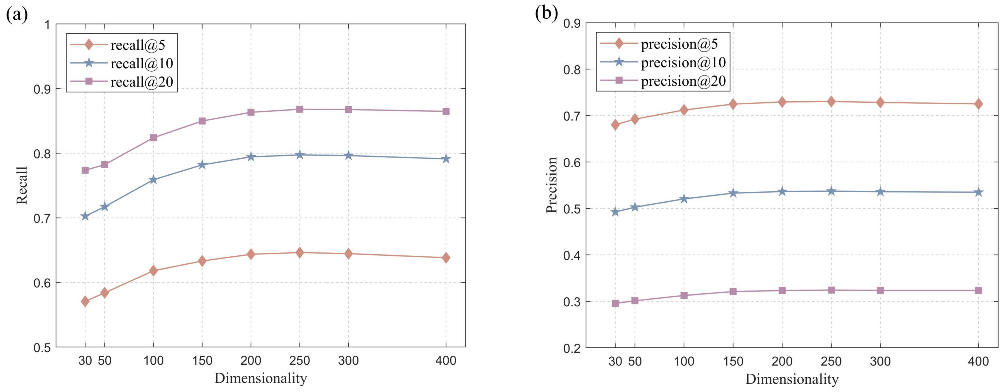

Supplementary Figure 2. The recall and precision values of RSML-GCN on the benchmark dataset with different dimensional settings.

Supplementary Figure 1 and Supplementary Figure 2 analyze the setting of parameter n. RSML-GCN projects drugs and diseases into the unified metric vector space, which characterizes drugs and diseases from different perspectives (dimensions). The latent vector dimension controls the complexity of the model, so an overly large n may lead to overfitting of the model, while a smaller n can reduce the representational capacity of the model. To verify the effect of spatial dimensionality on model performance, we conducted experiments on different vector dimension n settings. The AUPR and AUC of the RSML-GCN model on the benchmark dataset are shown in Supplementary Figure 1, and the performance of recalls and precisions in top-k prediction is shown in Supplementary Figure 2. We found that as the vector dimension n increased, RSML-GCN became more effective, and the model performance tended to be stable when n was greater than 200 and then gradually declined. Thus, the choice of dimension n in [200, 300] was appropriate. Here, we set the parameter n to 250.

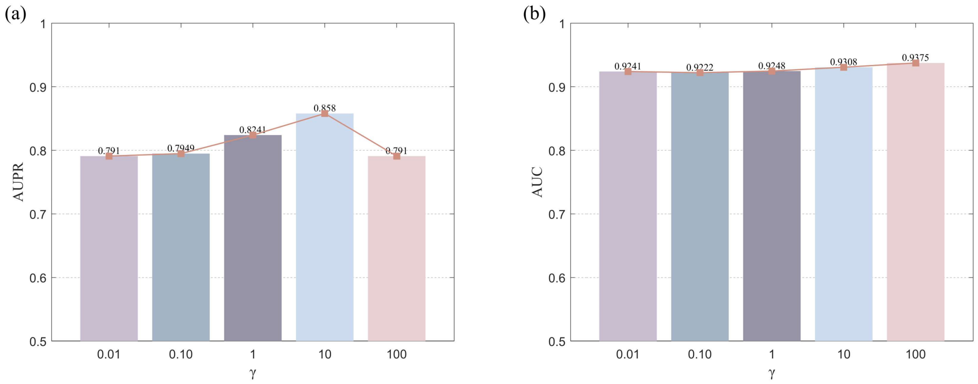

Supplementary Figure 3. The AUPR and AUC for different γ settings in model prediction.

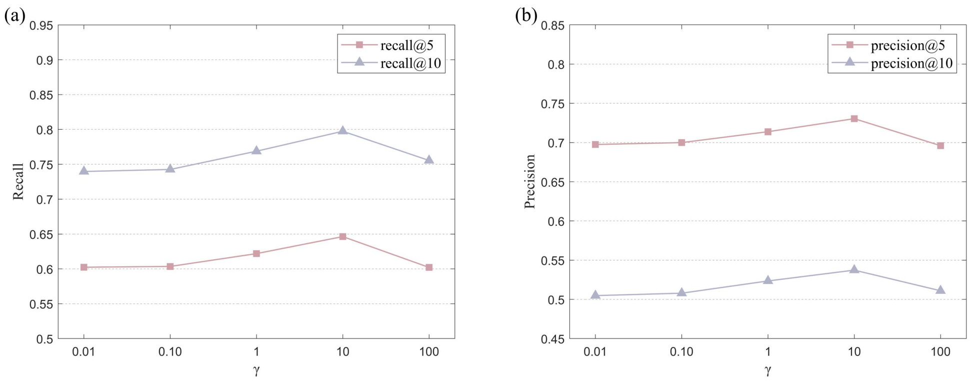

Supplementary Figure 4. The recall and precision values of model top-k predictions under different γ settings.

Supplementary Figure 3 and Supplementary Figure 4 analyze the setting of parameter γ. RSML-GCN introduces hyperparameters γ to control the strength of the margin in drug-centric and disease-centric learning. Here, we analyzed the impact of γ on the model performance and its settings on the dataset. When the latent space vector dimension was fixed to 250, we varied different values of γ. Supplementary Figure 3 shows the AUPR and AUC under different γ settings, demonstrating that the best performance of the model corresponded to 10. Supplementary Figure 4 further displays the results of RSML-GCN top-k prediction. It can be seen that when γ was taken as 10, the result was the best, and then the prediction performance of RSML-G0CN decreased with the change of γ. Therefore, γ was recommended to be set to 10.

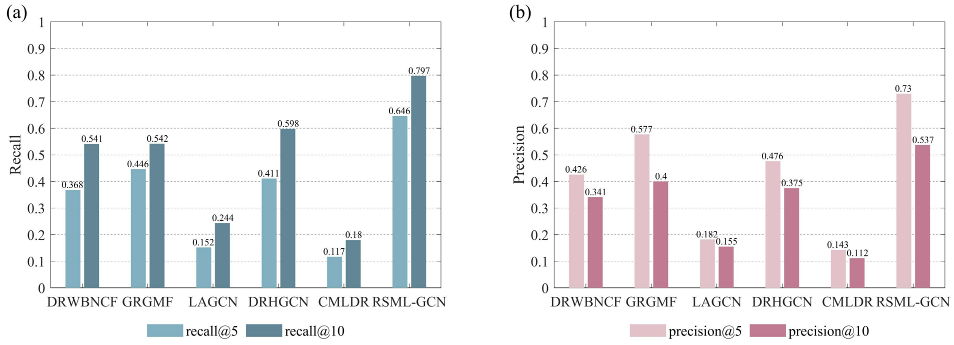

Supplementary Figure 5. The recall and precision values of different methods in top-k recommended drug indications on Cdataset.

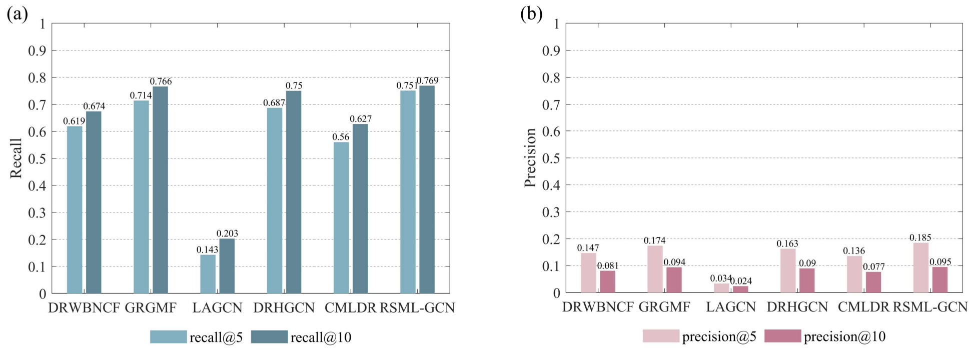

Supplementary Figure 6. The recall and precision values of different methods in top-k recommended drug indications on Fdataset.

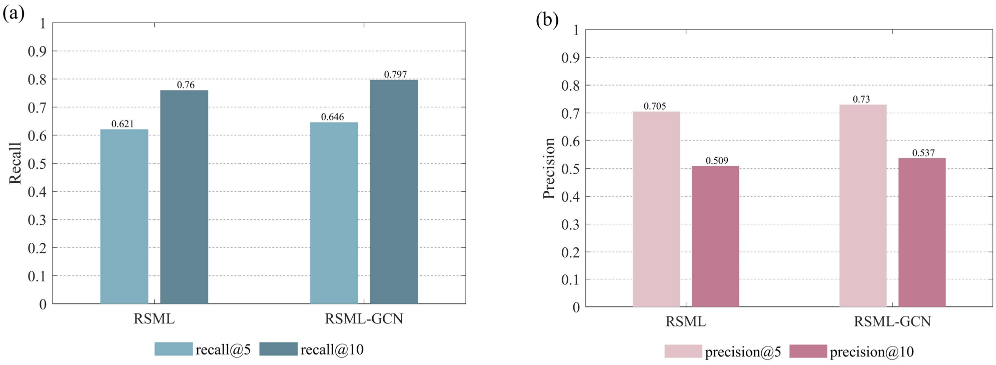

Supplementary Figure 7. Comparison of the recall and precision values of RSML with RSML-GCN in top-k recommended drug indications.

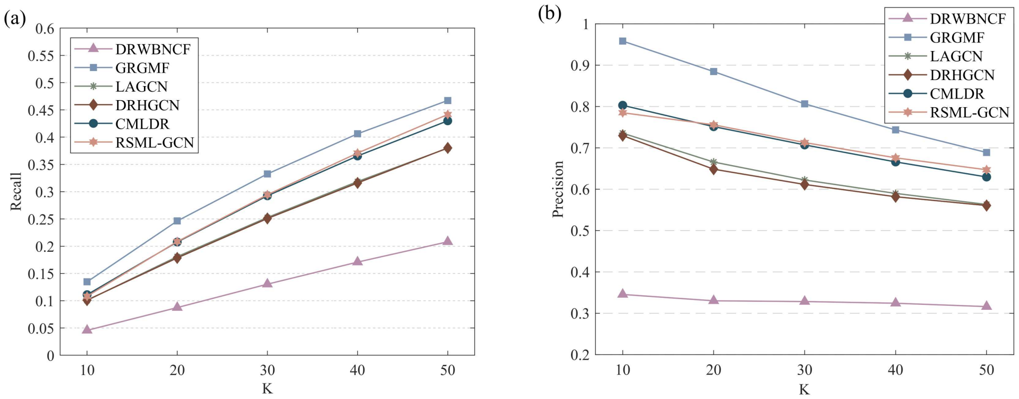

Supplementary Figure 8. The recall and precision values of RSML-GCN recommends top-k drugs for new diseases.

3 Supplementary References

Davis, A.P., Grondin, C.J., Johnson, R.J., Sciaky, D., King, B.L., McMorran, R., et al. (2016). The Comparative Toxicogenomics Database: update 2017. *Nucleic Acids Res.* 45(D1)**,** D972-D978. doi: 10.1093/nar/gkw838.

Deng, Y., Xu, X., Qiu, Y., Xia, J., Zhang, W., and Liu, S. (2020). A multimodal deep learning framework for predicting drug-drug interaction events. *Bioinformatics* 36(15)**,** 4316-4322. doi: 10.1093/bioinformatics/btaa501.

Flach, P., and Kull, M. (2015). Precision-recall-gain curves: PR analysis done right. *Advances in neural information processing systems* 28.

Gottlieb, A., Stein, G.Y., Ruppin, E., and Sharan, R. (2011). PREDICT: a method for inferring novel drug indications with application to personalized medicine. *Mol Syst Biol* 7**,** 496. doi: 10.1038/msb.2011.26.

Guo, Z., You, Z., Huang, D., Yi, H., Zheng, K., Chen, Z., et al. (2020). MeSHHeading2vec: a new method for representing MeSH headings as vectors based on graph embedding algorithm. *Brief. Bioinformatics* 22(2)**,** 2085-2095. doi: 10.1093/bib/bbaa037.

Law, V., Knox, C., Djoumbou, Y., Jewison, T., Guo, A.C., Liu, Y., et al. (2013). DrugBank 4.0: shedding new light on drug metabolism. *Nucleic Acids Res.* 42(D1)**,** D1091-D1097. doi: 10.1093/nar/gkt1068.

Lipscomb, C.E. (2000). Medical Subject Headings (MeSH). *Bull Med Libr Assoc* 88(3)**,** 265-266.

Wang, D., Wang, J., Lu, M., Song, F., and Cui, Q. (2010). Inferring the human microRNA functional similarity and functional network based on microRNA-associated diseases. *Bioinformatics* 26(13)**,** 1644–1650. doi: 10.1093/bioinformatics/btq241.

Wu, S., and Flach, P. (2005). "A scored AUC Metric for Classifier Evaluation and Selection", in: *Second workshop on ROC analysis in ML,* ,

Zhang, W., Yue, X., Lin, W., Wu, W., Liu, R., Huang, F., et al. (2018). Predicting drug-disease associations by using similarity constrained matrix factorization. *BMC Bioinformatics* 19(1)**,** 233. doi: 10.1186/s12859-018-2220-4.

Zhao, B., Hu, L., You, Z., Wang, L., and Su, X. (2021). HINGRL: predicting drug–disease associations with graph representation learning on heterogeneous information networks. *Brief. Bioinformatics* 23(1). doi: 10.1093/bib/bbab515.
